# Supplementary material for: Does social media information credibility influence social commerce purchase intention of skincare products? Evidence from Facebook
Source: PLoS One. 2025 Oct 22;20(10):e0334126. doi: 10.1371/journal.pone.0334126 (PMC12543200; doi:10.1371/journal.pone.0334126)
Supplement: Attachment — Submitted filename: Author_Response_to_Reviewers.docx [file pone.0334126.s003.docx]

**Point-by-Point Response to the Reviewers**

Dear Reviewers,

We sincerely thank you for your thoughtful and constructive comments on our manuscript. We greatly appreciate the time and effort you have invested in reviewing our work. Your insights have been instrumental in helping us improve the quality and clarity of the paper. We have carefully considered each comment and have revised the manuscript accordingly. All responses and revisions are detailed point by point in the following document ***(The mentioned line numbers are in the manuscript without track changes document).***

| **Reviewers’ Comment** | **Authors’ Response** |
| --- | --- |
| **Reviewer 1** | |
| The logical sequence of the abstract should be as 1) objectives, 2) methodology, 3) Findings, 4) conclusion and 5) implications. Thus, the authors should also rewrite the abstract in this sequence. | Thank you for your valuable and insightful feedback. In response we have rewrite the abstract to follow the recommended logical sequence:    (1) objectives, - To examine how social media information credibility factors (source credibility and electronic word-of-mouth (e WOM) credibility) influence consumers’ purchase intentions for skincare products on social commerce platforms (Facebook), considering the roles of trust in online communities and perceived risk.  (included in the manuscript) - **(Lines 88-90)**  “This study aims to examine how social media information credibility factors specifically source credibility and electronic word of mouth (e-WOM) credibility influence consumers’ purchase intentions for skincare products on Facebook.”  (2) Methodology- A structured survey collected data from 384 consumers who purchased skincare products via Facebook. The proposed model was tested using structural equation modeling (SEM). Source credibility and e WOM credibility were treated as independent variables; trust in online communities and perceived risk as mediators; and social commerce purchase intention as the dependent variable.  (included in the manuscript**)- (Lines 91-93)**  “Primary data were collected through a structured survey from 384 skincare purchasers who made their purchases via Facebook, and the model was tested using structural equation modelling (SEM)”  (3) findings- The SEM results indicate that source credibility, e WOM credibility, and trust in online communities each have a positive significant effect on social commerce purchase intention, while perceived risk has a negative effect. Furthermore, trust in online communities and perceived risk mediate the influence of information credibility on purchase intentions as well as trust also reducing perceived risk.  (Included in the manuscript**) : (Lines 93-99)**  “ Further, the results reveal that source credibility, e-WOM credibility, and trust in online communities positively influence social commerce purchase intention, while perceived risk has a negative effect. Trust in online communities also reduces perceived risk and mediates the relationship between information credibility and purchase intention. Hence, these findings highlight the pivotal roles of trust and risk perceptions in shaping online consumer behaviour in the social commerce space, especially within the skincare market”  (4) conclusion- Information credibility strongly shapes consumer trust and risk perceptions in social commerce. The findings underscore the pivotal role of building trust and minimizing perceived risk to encourage online purchase intentions, especially in the skincare domain.  (Included in the manuscript**) : (Lines 99-101)**  “The study emphasizes the need for businesses to leverage credible information sources and build trustworthy online communities to enhance consumer confidence and engagement.”  (5) implications- This study highlights the need for businesses to leverage credible information sources and foster trustworthy online communities to boost consumer confidence. It also contributes to the social commerce literature by providing insights from an emerging market (Sri Lanka) and identifies avenues for future research on social media information credibility.  (Included in manuscript**) : (Lines 101-104)**  *“Moreover, it contributes to the growing literature on social commerce by offering insights from an emerging market context Sri Lanka and suggests future research into broader dimensions of credibility and cultural comparisons to deepen the understanding of social commerce.”* |
| The authors did not establish the motivation, significance, and novelty of the undertaken study. The authors are suggested to improve this important factor in the "Introduction" section. The structure of the paper is also missing. | Thank you for your valuable and insightful feedback. In response. We have revised the "Introduction" section to clearly articulate the motivation, significance, and novelty of the study, particularly in the context of social media information credibility and purchase intention in Sri Lanka’s skincare sector. Additionally, we have added a brief outline at the end of the Introduction to clarify the structure of the paper.  In the introduction section we added paragraph about motivation of study.  As authors, we recognize the increasing influence of social media on consumer purchase behavior, particularly in the context of skincare products, which require a high level of trust and credible information. The motivation for this study lies in addressing this emerging trend by examining how credibility factors on social media shape purchase intentions. This is especially relevant for businesses in developing countries like Sri Lanka, where digital transformation is accelerating. Through this research, we aim to contribute insights that help these businesses develop effective digital strategies to build consumer trust and enhance performance in social commerce.  **(Included in manuscript) : (Lines 149-153)**  *“The motivation of this study stems from the growing reliance on social media platforms for purchase decisions and the critical need for credible information, particularly for sensitive products like skincare. Its significance lies in helping businesses in developing economies, such as Sri Lanka, build effective digital strategies to enhance consumer trust and drive social commerce success”.*  In the introduction section we added paragraph about the novelty of the study.  As authors the novelty of our study lies in its integrated examination of multiple dimensions of social media information credibility namely, source credibility and e-WOM credibility and their combined impact on social commerce purchase intention. We introduce perceived risk and trust in online communities as key mediators, offering a more holistic view of consumer decision-making processes. Unlike prior studies that have investigated these factors in isolation or outside a unified framework, our research presents a comprehensive model. By applying this model to the relatively unexplored context of Sri Lanka’s skincare market, we provide both theoretical contributions and actionable insights for practitioners in emerging digital economies.    **(Included in manuscript) : (Lines 171-177)**  *“Moreover, the novelty of this study lies in its integrated exploration of multiple social media information credibility, specifically source credibility and e-WOM credibility and their effects on social commerce purchase intention through the mediating roles of perceived risk and trust in online communities. Unlike previous research that examined these factors individually or outside of an integrated framework, this study combines them into a comprehensive model and applies it to the underexplored Sri Lankan skincare market within a social commerce context, providing fresh theoretical and practical insights”.*  In the introduction section we added paragraph about the significance of the study  The significance of this study lies in its potential to assist businesses in developing economies like Sri Lanka in crafting effective digital strategies. By understanding how credible information on social media builds consumer trust, businesses can better engage with consumers and enhance their performance in the growing field of social commerce.  **(Included in manuscript) : (Lines 151-153)**  *“Its significance lies in helping businesses in developing economies, such as Sri Lanka, build effective digital strategies to enhance consumer trust and drive social commerce success.”*  We have included a paragraph about Structure of the paper  **(Included in manuscript) : (Lines 183-187)**  *“The remaining sections are structured as follows: ‘Literature Review’, covers theories and prior findings on social media credibility and purchase intention Data and Methodology, explains the data collection and SEM approach. ‘Results and Discussion’, analysing the empirical findings and their interpretations, and “Conclusion,” provides recommendations and future research directions.”* |
| The literature should be presented in an audit form, and it should be linked with the objectives and research question. Moreover, the current and relevant citations should be added in introduction, review of literature and discussions sections. Moreover, the study has adopted a deductive approach, thus, the relevant theory should be integrated with the operational variables. The authors can have the useful insights by studying and citing the following papers: “Enhancing Competitiveness of E-commerce and the Online Retail Industry via Social Media: Evidence from an AI-Integrated Routine Model”, Journal of Competitiveness, 2024, Vol. 16 (4), 44–59. <https://doi.org/10.7441/joc.2024.04.03> “Social Media Marketing of Luxury Brands on Brand Equity, Customer equity and Customer Purchase Intention”, Amfiteatru Economic, 2023, Vol. 25 (62), 265–282. <https://doi.org/10.24818/EA/2023/62/265> “Effectiveness of online digital media advertising as a strategic tool for building brand sustainability: Evidence from FMCGs and Services sectors of Pakistan”, Sustainability, 2019, Vol. 11(12), 3436. <http://dxdoi.org/10.3390/su11123436> | We sincerely thank the reviewer for highlighting the gap in literature. In response, we thoroughly restructured the literature review section into clear thematic segments aligned with the research objectives and questions (Ali & Alvi, 2019). Specifically, we now explicitly show how source credibility, e WOM credibility, trust, and perceived risk have been treated in past studies, and how they relate to purchase intention in social commerce (Gondová & Madleňák, 2024).  We added following subsections under the literature review   - Source Credibility on perceived risk (Lines 217 – 227) - E WOM Credibility and Trust online community (Lines 248 – 258) - Perceived Risk and social commerce purchase intention (Lines 284 – 293) - Trust online community and perceived risk (Lines 305–312) and social commerce purchase intention (Lines 330 – 337) - Social Commerce Purchase Intention (Lines - We ensured that each variable is not only defined but justified through relevant recent citations from high-impact journals, as suggested (Pop, Dabija, & Iorga, 2023). - We also strengthened the theoretical underpinning by explicitly showing how the Elaboration Likelihood Model (ELM) justifies the selection of these constructs, detailing how they fit under the central and peripheral routes of persuasion. - Further references are added: - Gondová, K., & Madleňák, R. (2024). Enhancing competitiveness of e-commerce and the online retail industry via social media: Evidence from an AI-integrated routine model. *Journal of Competitiveness, 16*(4), 44–59. <https://doi.org/10.7441/joc.2024.04.03> - Pop, R. A., Dabija, D. C., & Iorga, A. M. (2023). Social media marketing of luxury brands on brand equity, customer equity, and customer purchase intention. *Amfiteatru Economic, 25*(62), 265–282. <https://doi.org/10.24818/EA/2023/62/265> - Ali, B., & Alvi, M. H. (2019). Effectiveness of online digital media advertising as a strategic tool for building brand sustainability: Evidence from FMCGs and services sectors of Pakistan. *Sustainability, 11*(12), 3436. <https://doi.org/10.3390/su11123436>   Thank you for your valuable feedback. In response, we have added new and relevant citations in the introduction section to better support the identified research gap and provide a stronger foundation for the study’s motivation and context.   - Ahmed R, Berchtold G, Streimikiene D, Soomro DR. Questionnaire: Effectiveness of online digital media advertising as a strategic tool for building brand sustainability: Evidence from fmcgs and services sectors of pakistan. Sustainability 2019;11(12):3436. <https://doi.org/10.3390/su11123436> **(**Lines 922-925) - Duan X, Chen C-N, Shokouhifar M. Impacts of social media advertising on purchase intention and customer loyalty in e-commerce systems. ACM Trans Asian Low-Resour Lang Process. 2024;23(8):1-15. <https://doi.org/10.1145/3613448>   (Lines 897-900)   - Ahmed R, Zaidi E, Alam SH, Streimikiene D, Parmar V. Effect of social media marketing of luxury brands on brand equity, customer equity and customer purchase intention. Amfiteatru Economic. 2023;25(62):265-82. <https://doi.org/10.24818/EA/2023/62/265> (Lines 914-916) - Govender KK, Yavisha R. The impact of social commerce on the purchase intentions of millennials using facebook. Innovative Marketing. 2023;19:223-35. <https://doi.org/10.21511/im.19(2).2023.18> (Lines 940-942) |
| The methodology should contain the research design, measures, data collection method, and data analysis techniques in separate headings along with the existing sub-headings. | **Thank you for your suggestion.** We have restructured the **Methodology** section to include separate headings for **Research Design, Measures,** **Data Collection Method,** and **Data Analysis Techniques,** as recommended. This revised structure clearly outlines the methodology's key aspects and enhances the manuscript's clarity and readability.   **Revisions that we addressed:**  • **Research Design (Lines 429–441):** A clear description of the **research design,** including the quantitative approach, cross-sectional time horizon, and convenience sampling method used in the study.  • **Measures (Lines 463–470):** Detailed information on the **constructs** measured (e WOM credibility, source credibility, trust in online community, perceived risk, and purchase intention), along with the **measurement scales** used and references to the original sources of these scales.  • **Data Collection Method (Lines 474–493):** A separate section explaining the **sampling procedure**, including the use of **Facebook** as the data collection platform, and details about the **sample size** and demographic profile of participants.  • **Data Analysis Techniques (Lines 504 –519): A** clear explanation of the **PLS-SEM** method, including the use of **SmartPLS 4** for analysis, the validation of **measurement models**, and the evaluation of **structural models** with appropriate statistical tests. |
| The PLS-SEM analysis was performed; however, the researchers should have validated the measurement model (outer model) using the reliability and validity analyses using factor loadings, Cronbach’s alpha, composite reliability, average variance extracted for reliability and convergent validity analyses. Similarly, for the discriminant validity the researchers should have used HTMT correlation matrix, Fornell-Larcker criterion, and cross loading analyses. For the validation of structural model (inner model), the coefficient of variation (R2), effect size (f2), predictive power (Q2), Model fitness indices for measurement and structural model besides the coefficient path analysis should be used. The Tables and figures of measurement model and structural model should also be presented. The detailed insights of PLS-SEM modeling could be taken by studying and citing the following paper: A Comparative Analysis of Multivariate Approaches for Data Analysis in Management Sciences,” E a M: Ekonomie a Management, Vol. 27 (1), 2024, 192–210. <https://doi.org/10.15240/tul/001/2024-5-001> | Thank you for your valuable and insightful feedback. In response, we have revised the manuscript extensively to ensure full compliance with the PLS-SEM guidelines for both the measurement model and the structural model.   **Measurement Model (Lines 530–578)**:  **Reliability and Validity (Lines 537–551)**:   - Indicator reliability was confirmed using factor loadings, all of which exceed the 0.6 threshold (Hair et al., 2021; Sarstedt et al., 2017). - Internal consistency was assessed using Cronbach’s Alpha and Composite Reliability (CR), both of which exceed 0.70 for all constructs. - Average Variance Extracted (AVE) values for all constructs were above 0.50, confirming convergent validity.   These results are reported in Table 3.  **Discriminant Validity (Lines 552–578)**:  We assessed discriminant validity using the three recommended techniques:   - Cross-loadings - Fornell-Larcker Criterion - HTMT ratio (Henseler et al., 2015)   The values met all threshold criteria, indicating acceptable discriminant validity. This is presented in Table 4, 5 and 6.  **Structural Model (Lines 580–661)**:  We then assessed the inner model using:   - **R² values** for the dependent variables, which demonstrate substantial explanatory power (Chin, 2010) - **Q² values** obtained through blindfolding to test predictive relevance - **f² effect sizes** to determine the relative impact of exogenous constructs - **Model Fit Indices**, including SRMR and NFI, which confirmed the model's goodness of fit (Hu & Bentler, 1999; Henseler et al., 2014).   These are reported in Table 7, 8 and 9.  The **path coefficients** and their significance (using 5000-sample bootstrapping) are also presented in Table 10. Table 11 shows significant mediation effects, supported by confidence intervals and t-values.  **Figures Added**:   - Figure 3 (Line 531) illustrates the measurement model (outer model). - Figure 4 (Line 615) presents the structural model (inner model).   **Finally, we have studied and cited the paper you recommended:** • Majeed, S., & Rana, T. (2024). *A Comparative Analysis of Multivariate Approaches for Data Analysis in Management Sciences*. *E a M: Ekonomie a Management*, 27(1), 192–210. <https://doi.org/10.15240/tul/001/2024-5-001>  (Line 564)  These enhancements ensure rigorous PLS-SEM model validation and enhance the robustness of the manuscript.  **The following is a list of DOIs for the citations that were added in the lines above.**   - Hair, J. F., Hult, G. T. M., Ringle, C. M., & Sarstedt, M. (2021). *A Primer on Partial Least Squares Structural Equation Modeling (PLS-SEM)* (3rd ed.). Sage Publications.<https://us.sagepub.com/en-us/nam/a-primer-on-partial-least-squares-structural-equation-modeling-pls-sem/book270548> - Sarstedt, M., Hair, J. F., Ringle, C. M., Thiele, K. O., & Gudergan, S. P. (2016). Estimation issues with PLS and CBSEM: Where the bias lies! *Journal of Business Research*, 69(10), 3998–4010. <https://doi.org/10.1016/j.jbusres.2016.06.007> - Henseler, J., Ringle, C. M., & Sarstedt, M. (2015). A new criterion for assessing discriminant validity in variance-based structural equation modeling. *Journal of the Academy of Marketing Science*, 43(1), 115–135. <https://doi.org/10.1007/s11747-014-0403-8> - Chin, W. W. (2010). How to write up and report PLS analyses. In V. Esposito Vinzi, W. Chin, J. Henseler & H. Wang (Eds.), *Handbook of Partial Least Squares* (pp. 655–690). Springer. <https://doi.org/10.1007/978-3-540-32827-8_29> - Hu, L. T., & Bentler, P. M. (1999). Cutoff criteria for fit indexes in covariance structure analysis: Conventional criteria versus new alternatives. *Structural Equation Modeling: A Multidisciplinary Journal*, 6(1), 1–55. <https://doi.org/10.1080/10705519909540118> - Henseler, J., Hubona, G., & Ray, P. A. (2016). Using PLS path modeling in new technology research: Updated guidelines. *Industrial Management & Data Systems*, 116(1), 2–20. <https://doi.org/10.1108/IMDS-09-2015-0382> |
| Please follow the sequence as: the discussions section should be added after the results. Followed by theoretical and practical implications, and then limitations and potential areas of future studies. | **Thank you for your valuable suggestion.** We have restructured the manuscript to align with your recommended sequence: **Discussion, Theoretical and Practical Implications,** followed by **Limitations and Future Research Directions.** This revised order enhances the logical flow of the manuscript, ensuring a smooth transition from **results** to **implications** and finally to **future research suggestions.**  **Revisions Made:**  • **Discussion Section (Lines 662–752):**  We have added a comprehensive **discussion** section, interpreting and contextualizing the results with reference to existing literature. This includes a comparison of our findings with previous studies, highlighting similarities and differences.   • **Theoretical (Lines 754-775) and Managerial Implications (Lines 776-798):**  After the **Discussion**, we provide insights into the theoretical and practical implications of our findings for both **social commerce theory** and **skincare marketing strategies.**  • **Limitations and Future Research Directions (Lines 825-874):**  We conclude the manuscript by addressing the **limitations** of the study, such as the **convenience sampling method,** and proposing **future research** areas, focusing on **cross-cultural comparisons, platform diversity,** and the **integration of emerging technologies.** |
| The conclusion needs further elaboration and adds at the end of the paper; the conclusion is always one step ahead of the findings. | **Thank you for your valuable suggestion.** We have revised the **Conclusion** section to provide further elaboration and ensure it is positioned as "one step ahead" of the findings. The revised conclusion now not only summarizes the key findings of the study but also provides insights into how these findings contribute to the broader context of **social commerce** and **skincare marketing.** Additionally, we have included **practical implications** for businesses, highlighting how the findings can inform **marketing strategies** and **consumer trust-building** initiatives in **social commerce** platforms.  **Revisions Made:**  • **Expanded Conclusion (Lines 799–824):**  We have elaborated on the **implications** of the findings, both theoretically and practically. We discuss how the findings can influence **future research** directions and offer recommendations for **skincare brands** to improve **consumer trust** through **platforms like Facebook**. We have also provided suggestions for leveraging **blockchain technology** and **AI-driven recommendation systems** to enhance **consumer trust** and **purchase intention**. |

| **Reviewers’ Comment** | **Authors’ Comment** |
| --- | --- |
| **Reviewer 2** | |
| The study uses source credibility, e WOM, trust in online communities, and perceived risk as predictors of social commerce purchase intention, but it lacks a clear justification of the selection of these specific variables. **Recommendation:** Provide a stronger theoretical rationale for why these specific factors were chosen and how they relate to existing models in consumer trust and e-commerce research. | We sincerely thank the reviewer for highlighting this important gap. Based on the feedback, we have strengthened the justification of the selection of the key variables under the Conceptual Model section (Lines 400 -422)  **Specifically, we now explain that:**   - Source credibility and eWOM (electronic word of mouth) are peripheral cues under the ELM (Petty & Cacioppo, 1986), shaping attitudes and purchase intentions especially when consumers are not deeply engaged in central processing. - Trust in online communities serves as a crucial mediator, grounded in the trust transfer theory (Stewart, 2003), where trust in a platform or peer group enhances trust in sellers and the purchasing process. - Perceived risk is included following the risk-based trust model (Pavlou, 2003), where perceived risk negatively impacts trust and purchase decisions in online commerce settings.   We also integrate recent empirical studies that validate these constructs as central predictors of online consumer behavior (Gondová & Madleňák, 2024; Pop, Dabija, & Iorga, 2023; Ali & Alvi, 2019). This expanded section clarifies why these variables are theoretically appropriate and strengthens the study’s foundation.   - Petty, R. E., & Cacioppo, J. T. (1986). *The elaboration likelihood model of persuasion.* Advances in Experimental Social Psychology, 19, 123–205. - Stewart, K. J. (2003). Trust transfer on the World Wide Web. *Organization Science, 14*(1), 5–17. <https://doi.org/10.1287/orsc.14.1.5.12810> - Pavlou, P. A. (2003). Consumer acceptance of electronic commerce: Integrating trust and risk with the technology acceptance model. *International Journal of Electronic Commerce, 7*(3), 101–134. https://doi.org/10.1080/10864415.2003.11044275 - Gondová, K., & Madleňák, R. (2024). Enhancing competitiveness of e-commerce and the online retail industry via social media: Evidence from an AI-integrated routine model. *Journal of Competitiveness, 16*(4), 44–59. <https://doi.org/10.7441/joc.2024.04.03> - Pop, R. A., Dabija, D. C., & Iorga, A. M. (2023). Social media marketing of luxury brands on brand equity, customer equity, and customer purchase intention. *Amfiteatru Economic, 25*(62), 265–282. <https://doi.org/10.24818/EA/2023/62/265> - Ali, B., & Alvi, M. H. (2019). Effectiveness of online digital media advertising as a strategic tool for building brand sustainability: Evidence from FMCGs and services sectors of Pakistan. *Sustainability, 11*(12), 3436. <https://doi.org/10.3390/su11123436> |
| The Elaboration Likelihood Model (ELM) is briefly mentioned, but the integration of this theory into the study is unclear.  **Recommendation:** Clearly explain how the ELM framework guides the study and how different credibility factors align with the central vs. peripheral route of persuasion. | **Thank you for your valuable comment.** We have expanded on the explanation of how the **Elaboration Likelihood Model (ELM)** is integrated into the study. The ELM serves as the theoretical framework that guides the **credibility** factors influencing **social commerce purchase intention (SCPI).** We have clarified how **central** and **peripheral routes** of persuasion are relevant to the credibility factors in the context of **e WOM, source credibility,** and **social media information credibility.**  **Revisions we addressed as below:**  • **Integration of ELM Framework (Lines 839–844):**  We have added a comprehensive section explaining the role of **ELM** in this study. The **ELM** helps differentiate between two types of persuasion processes: the **central route** and the **peripheral route.** These routes are relevant to **social commerce** because they explain how consumers process and evaluate information before making a purchase decision. In our study, we argue that **e WOM credibility** and **source credibility** are factors that influence the central route of persuasion. When consumers are highly motivated and able to process information carefully, they focus on the **quality and reliability** of information, which enhances **purchase intention**. On the other hand, **social media information credibility** and **trust in the online community** are factors that influence the **peripheral route** of persuasion, where consumers are less involved and rely more on **heuristics** (e.g., trust in community norms or the source’s reputation) rather than careful evaluation of the information itself. In this section, we have provided a clearer explanation of how **credibility factors** align with the **central** and **peripheral routes** of persuasion in the **ELM** framework. For instance, consumers might be persuaded by the **central route** if they trust the detailed reviews or the **expertise** of the source (e.g., skincare professionals or influencers with a strong reputation). Alternatively, the **peripheral route** may be triggered by **social media cues**, such as **likes** and **comments** on posts or the general popularity of a product in an online community, which makes consumers more likely to purchase due to social influence rather than a deep analysis of the product’s benefits. |
| The manuscript states that Facebook was selected as the primary social commerce platform but does not provide a strong justification for excluding other platforms (e.g., Instagram, TikTok, WeChat, etc.), which also play significant roles in the skincare industry. **Recommendation:** Explain why Facebook was chosen over other platforms and discuss potential platform-specific biases in consumer behavior. | We sincerely thank the reviewer for this thoughtful observation. We have now expanded the justification for selecting Facebook (Lines 450-459) under the Methodology section (Research Design) to clarify why it was chosen as the primary social commerce platform for this study.  **Specifically, we explain that:**   - Facebook is the most widely used social media platform in Sri Lanka with over 7 million active users, significantly surpassing Instagram and TikTok in market penetration (Statista, 2024). - Facebook offers integrated social commerce features (such as Facebook Marketplace, Shop, and in-app purchase tools) that are well-established and frequently used by local skincare sellers, unlike Instagram and TikTok, which are more focused on visual content sharing and influencer marketing rather than direct social commerce transactions (Gondová & Madleňák, 2024). - The consumer demographics on Facebook in Sri Lanka, especially among skincare purchasers, skew toward the target group studied (young adults and women aged 18–35), making it a strategic and representative choice (Pop, Dabija, & Iorga, 2023).   We also acknowledge the platform-specific bias as a study limitation, noting that consumer behavior on Instagram or TikTok may differ, and recommend future research to conduct cross-platform comparative studies to broaden generalizability.   - Statista. (2024). *Social media usage in Sri Lanka - Statistics & facts*. Retrieved from <https://www.statista.com> - Gondová, K., & Madleňák, R. (2024). Enhancing competitiveness of e-commerce and the online retail industry via social media: Evidence from an AI-integrated routine model. *Journal of Competitiveness, 16*(4), 44–59. <https://doi.org/10.7441/joc.2024.04.03> - Pop, R. A., Dabija, D. C., & Iorga, A. M. (2023). Social media marketing of luxury brands on brand equity, customer equity, and customer purchase intention. *Amfiteatru Economic, 25*(62), 265–282. <https://doi.org/10.24818/EA/2023/62/265> |
| The sampling method is described as convenience sampling, which limits the generalizability of findings. **Recommendation:** Acknowledge the limitations of convenience sampling more explicitly and discuss how this affects the applicability of the results to broader populations. | We sincerely thank the reviewer for pointing out this limitation and appreciate the suggestion. In response, we have explicitly acknowledged the limitations of convenience sampling in the Methodology section (Sampling Method) and have discussed the implications for the generalizability of the results (Lines 827-831).  **Specifically, we explain:**   - Convenience sampling was employed due to the limited access to a larger and more diverse population of skincare purchasers in Sri Lanka. The practical constraints of time, budget, and data availability made this the most feasible approach. - However, we fully recognize that convenience sampling introduces selection bias, as the sample may not accurately represent the entire population of skincare purchasers in Sri Lanka. The participants were primarily selected based on their availability and willingness to respond, which may not account for broader demographic or regional variations. - We have now highlighted the impact of this sampling method on the external validity of the study's findings, acknowledging that while the results are insightful for understanding social commerce behaviors in the specific context of Sri Lankan Facebook users, they cannot be generalized to other populations or countries. - Future studies using probability sampling techniques (such as stratified or random sampling) are recommended to increase the generalizability of the findings across diverse consumer segments and geographies. |
| The study examines Sri Lankan consumers, but it does not discuss cultural differences that might affect trust in online reviews and information credibility. **Recommendation:** Include a discussion on cultural factors that may influence social commerce behaviors, particularly in comparison to global markets. | We greatly appreciate the reviewer’s valuable suggestion. In response, we have expanded the discussion of cultural factors that could influence trust in online reviews and information credibility in Sri Lanka. This addition has been integrated into the Discussion section, specifically in relation to how Sri Lankan consumer behavior may differ from global trends (Lines 712-753).  **In this revised section, we discuss:**   - Cultural dimensions (Hofstede, 2001) such as collectivism and uncertainty avoidance, which are prevalent in Sri Lankan society, and how these cultural traits might shape consumer trust and behavior in social commerce. In more collectivist cultures, consumers tend to rely more on peer recommendations and social influences (like eWOM) than in individualistic societies, which may explain the higher trust placed in online community feedback in Sri Lanka compared to Western markets (Gondová & Madleňák, 2024). - Sri Lankans' strong preference for family and social ties influences their trust in social commerce, making online communities a significant source of information credibility. This contrasts with individualistic cultures, where consumers are more likely to prioritize personal research and brand reputation over peer opinions (Pop, Dabija, & Iorga, 2023). - Religious and traditional values in Sri Lanka may also impact perceptions of risk and trust in online platforms. Sri Lankan consumers may show higher perceived risk in adopting new technologies for purchase, especially in the context of skin care products, due to concerns about authenticity and potential fraud. This behavior could differ from global markets, where younger and more tech-savvy consumers may have a lower perceived risk in purchasing from online platforms (Ali & Alvi, 2019). - Hofstede, G. (2001). Culture’s consequences: Comparing values, behaviors, institutions, and organizations across nations. Sage Publications. - Gondová, K., & Madleňák, R. (2024). Enhancing competitiveness of e-commerce and the online retail industry via social media: Evidence from an AI-integrated routine model. Journal of Competitiveness, 16(4), 44–59. <https://doi.org/10.7441/joc.2024.04.03> - Pop, R. A., Dabija, D. C., & Iorga, A. M. (2023). Social media marketing of luxury brands on brand equity, customer equity, and customer purchase intention. Amfiteatru Economic, 25(62), 265–282. <https://doi.org/10.24818/EA/2023/62/265> - Ali, B., & Alvi, M. H. (2019). Effectiveness of online digital media advertising as a strategic tool for building brand sustainability: Evidence from FMCGs and services sectors of Pakistan. Sustainability, 11(12), 3436. <https://doi.org/10.3390/su11123436> |
| While structural equation modeling (SEM) is used, the results section lacks sufficient detail on model validation, fit indices, and robustness checks. **Recommendation:**   - Provide model fit indices (e.g., CFI, RMSEA, SRMR) to confirm the suitability of the proposed model. - Discuss any modification indices or respecifications made to improve model fit. - Justify why Smart PLS was chosen over other SEM techniques (e.g., AMOS, LISREL). | Thank you for this constructive suggestion. In the revised manuscript, we have incorporated the following updates to address all aspects of your comment:  **Model Fit Indices (Lines 600–610, Table 9):**   - We have reported model fit statistics for the PLS-SEM model, including Standardized Root Mean Square Residual (SRMR), Normed Fit Index (NFI), and Chi-square statistics, which are widely accepted in PLS-SEM literature. SRMR value was below 0.08, and NFI was above 0.80, indicating an acceptable model fit, this approach follows recommendations by Henseler et al. (2014) and Hair et al. (2021). - Please note that CFI and RMSEA are not typically applicable in variance-based SEM like Smart PLS.   **Respecifications :**  We checked for potential model misspecifications using modification indices. However, no major respecifications were made as the measurement model met all the reliability and validity thresholds, and the initial model fit was satisfactory.  **Software Justification (Lines 510–517):**  We have added a justification for the use of Smart PLS 4 over covariance-based SEM tools such as AMOS or LISREL. The justification is based on:   - The exploratory nature of our research, - The non-normal distribution of our data, and - The presence of a complex mediation model with multiple latent constructs, which is better handled through variance-based SEM.   *“Smart PLS 4 was chosen over covariance-based SEM tools such as AMOS or LISREL due to the exploratory nature of this study, the non-normal distribution of the dataset, and the complexity of the research model, which includes multiple mediation paths and latent constructs. Variance-based SEM, such as PLS-SEM, is more suitable for prediction-oriented research and models with a higher level of complexity. Moreover, PLS-SEM is robust to violations of multivariate normality.* *These choices are supported by* (Hair & Alamer, 1988) *and* (Ahmed & Streimikiene, 1979) *emphasized the practical advantages of PLS-SEM in similar research contexts.”*  These improvements strengthen the methodological rigor and transparency of the results section.  **The following is a list of DOIs for the citations that were added in the lines above.**   - Hair, J. F., Hult, G. T. M., Ringle, C. M., & Sarstedt, M. (2021). *A Primer on Partial Least Squares Structural Equation Modeling (PLS-SEM)* (3rd ed.). Sage Publications.<https://us.sagepub.com/en-us/nam/a-primer-on-partial-least-squares-structural-equation-modeling-pls-sem/book270548> - Hair J, Alamer A. Partial least squares structural equation modeling (pls-sem) in second language and education research: Guidelines using an applied example. Research Methods in Applied Linguistics. 2022;1(3):100027. <https://doi.org/10.1016/j.rmal.2022.100027> - **Henseler, J., Ringle, C. M., & Sarstedt, M. (2014).** A new criterion for assessing discriminant validity in variance-based structural equation modeling. Journal of the Academy of Marketing Science, **43**(1), 115–135. <https://doi.org/10.1007/s11747-014-0403-8> - Ahmed RR, Streimikiene D, Streimikis J, Siksnelyte-Butkiene I. A comparative analysis of multivariate approaches for data analysis in management sciences. E+M Ekonomie a Management. 2024;27(1):192-210. <https://doi.org/10.15240/tul/001/2024-5-001> |
| The mediation effects of trust and perceived risk are mentioned, but specific indirect effect values and confidence intervals are missing. **Recommendation**: Include bootstrap confidence intervals for mediation effects to ensure statistical rigor. | Thank you for this helpful recommendation. We have addressed the concern by including a detailed report of the indirect effects, along with bootstrap confidence intervals, using Smart PLS bootstrapping (5000 subsamples) procedure.  **Additions made in the revised manuscript:**  **Lines 612–614:**   - We have reported the indirect path coefficients, their t-values, p-values, and bias-corrected 95% confidence intervals for all mediating effects involving Trust in Online Community (TOC) and Perceived Risk (PR). These are presented in Table 11. - This includes mediation from Source Credibility (SC) and eWOM Credibility (EW) to Social Commerce Purchase Intention (SCPI). These are presented in Table 11. - The significance of indirect effects was confirmed as the confidence intervals did not include zero, demonstrating the presence of mediation.   Including these confidence intervals strengthens the robustness and statistical validity of our mediation analysis, in line with current SEM reporting standards. |
| The discussion largely reiterates the results without providing critical analysis or industry implications. **Recommendation:** Expand on how businesses can leverage these findings. For example, how can skincare brands improve consumer trust using blockchain verification or AI-driven recommendation systems? | We appreciate the reviewer’s valuable suggestion and have taken it into account. In response, we have significantly expanded the Discussion section to provide critical analysis and industry implications of our findings. We now include a detailed exploration of how businesses, particularly skincare brands, can leverage the insights from our study to improve consumer trust and social commerce purchase intention (**Lines 732-742**).  **Specifically, we discuss:**  Blockchain Technology for Trust Verification: We propose that skincare brands can use blockchain technology to verify product authenticity, which is a significant concern for consumers in the skincare industry (Ali & Alvi, 2019). With the rise of counterfeit products, integrating blockchain into the supply chain allows consumers to verify the legitimacy of the products they are purchasing by scanning QR codes that provide real-time information on the product’s origin, ingredients, and quality assurance certifications. This could substantially reduce perceived risk and increase trust in online reviews and eWOM, aligning with findings from our study that trust in online communities positively impacts purchase intention.  AI-driven Recommendation Systems: We also highlight the potential for AI-driven recommendation systems to enhance personalized consumer experiences. By analyzing consumer behavior and preferences, AI tools can recommend products more effectively, improving user engagement and trust in the platform. For instance, skincare brands could use AI to recommend personalized skincare routines based on individual preferences and skin types. This would increase consumer trust and help alleviate concerns regarding the effectiveness and safety of products (Duan, Chen, & Shokouhifar, 2024). Such systems can be integrated with eWOM to strengthen recommendations from trusted online communities, ensuring higher purchase intentions.  Influence of Social Media Influencers: Additionally, we discuss how businesses can collaborate with influencers to enhance trust in e WOM. In the skincare industry, influencers play a key role in promoting products, but businesses can ensure the credibility of their messages by partnering with trusted influencers who align with their brand values. By doing so, businesses can capitalize on the power of social media to create genuine, trust-based interactions with consumers.  Building Community Trust: We emphasize the importance of creating a community-oriented platform where customers can share their skincare experiences, reviews, and product recommendations. Brands that engage with and foster their communities on social media (especially Facebook, as indicated in our study) can improve the credibility of their products and enhance consumer trust by showing transparency, responding to feedback, and providing detailed product information.  These strategies are based on our findings that trust in online communities and eWOM have a significant impact on purchase intention, and perceived risk negatively impacts this relationship.   - Ali, B., & Alvi, M. H. (2019). Effectiveness of online digital media advertising as a strategic tool for building brand sustainability: Evidence from FMCGs and services sectors of Pakistan. *Sustainability, 11*(12), 3436. <https://doi.org/10.3390/su11123436> - Duan, X., Chen, C. N., & Shokouhifar, M. (2024). Impacts of social media advertising on purchase intention and customer loyalty in e-commerce systems. *ACM Transactions on Asian and Low-Resource Language Information Processing, 23*(8), 1-15. |
| The manuscript does not compare findings to previous studies in social commerce or skincare marketing. **Recommendation:** Provide a deeper comparative discussion with similar studies in Asia, Europe, and North America to highlight differences and similarities. | We thank the reviewer for this insightful suggestion. In response, we have expanded the comparative analysis in the Discussion section to provide a deeper comparison between our findings and existing studies in the field of social commerce and skincare marketing across Asia, Europe, and North America (Lines 723-731).  **Specifically, we have made the following improvements:**   1. Comparison with Studies in Asia: We compared our findings with studies conducted in Asia, particularly in regions with strong social commerce markets such as China and South Korea. For instance, in a study by Govender & Yavisha (2023) on millennial consumers in South Africa (who use Facebook as a major platform for skincare purchases), the researchers found that trust in online reviews has a significant positive impact on purchase intention. Our study aligns with this finding, emphasizing that trust in online communities significantly impacts purchase intention in Sri Lanka as well. However, our study is unique in highlighting how perceived risk in Sri Lanka influences consumer behavior, which differs from findings in China, where social commerce behavior is shaped more by peer pressure and less by risk concerns (Duan, Chen, & Shokouhifar, 2024). This cultural difference can be attributed to the individualistic tendencies in China, as opposed to the more collectivist culture in Sri Lanka. 2. Comparison with Studies in Europe: We also compared our findings with those from Europe, where luxury brands are particularly active in social commerce. According to Pop, Dabija, & Iorga (2023), European consumers tend to rely more on brand reputation and personal experience rather than e WOM for purchasing luxury skincare products. Our study diverges here, showing that in Sri Lanka, eWOM and social media influencers have a more significant impact on trust and purchase intention. This suggests that while brand reputation is important globally, Sri Lankan consumers are more influenced by peer recommendations on social media platforms like Facebook. 3. Comparison with Studies in North America: In the context of North America, we compared our results with studies like Kumar & Sharma (2020), who found that social media advertising on platforms like Instagram plays a dominant role in shaping purchase intention in the U.S. market. Our study, however, provides a contrasting perspective, focusing on Facebook as the preferred platform in Sri Lanka, where older consumers may feel more comfortable, as opposed to younger users in the U.S. who are more likely to engage with Instagram. Moreover, perceived risk plays a more significant role in the Sri Lankan market, as evidenced by our findings, where consumers express more concerns about the authenticity of products, a pattern that was less prominent in U.S. studies. 4. Cultural Differences: Additionally, we highlighted cultural differences across regions in terms of trust in online reviews. For example, studies in North America often report that younger generations show lower perceived risk and greater trust in eWOM, especially through influencers (Govender & Yavisha, 2023). In contrast, our study emphasizes how Sri Lankan consumers, particularly older generations, may be more cautious and skeptical about eWOM due to cultural influences, such as stronger familial ties and traditional values.  - Duan, X., Chen, C. N., & Shokouhifar, M. (2024). Impacts of social media advertising on purchase intention and customer loyalty in e-commerce systems. *ACM Transactions on Asian and Low-Resource Language Information Processing, 23*(8), 1-15. - Govender, K. K., & Yavisha, R. (2023). The impact of Social Commerce on the purchase intentions of millennials using Facebook. *Innovative Marketing, 19*(2), 223. - Kumar, A., & Sharma, N. K. (2020). Impact of social media on consumer purchase intention: A developing country perspective. In *Handbook of Research on the Role of Human Factors in IT Project Management* (pp. 260-277). IGI Global Scientific Publishing. - Pop, R. A., Dabija, D. C., & Iorga, A. M. (2023). Social media marketing of luxury brands on brand equity, customer equity, and customer purchase intention. *Amfiteatru Economic, 25*(62), 265–282. <https://doi.org/10.24818/EA/2023/62/265> |

| **Reviewers’ Comment** | **Authors’ Comment** |
| --- | --- |
| **Reviewer 3** | |
| For the literature review, authors should refer to more recent research. The current references are not up to date. Authors need to refer to more ISI/Scopus research work instead of the online/conference resources. | We thank the reviewer for the constructive feedback. In response to this suggestion, we have updated the literature review by incorporating more recent and high-quality sources from ISI/Scopus indexed journals. We understand the importance of using peer-reviewed articles from recognized academic journals to provide a solid theoretical foundation for the study.  The updated references now include more recent research on social commerce, eWOM, and trust in online communities, which have been published in top-tier journals and offer relevant and current insights into the field. This includes studies on consumer behavior, social media marketing, purchase intention, and platform-specific dynamics, which are more aligned with the scope of our study.  In addition, we have ensured that we removed references from online sources and conference proceedings that may not have the same academic rigor as Scopus-indexed journal articles. This aligns with the reviewer's recommendation to rely on higher-quality research to better support the findings and discussions in our manuscript.  The updated literature review section now features recent research published in journals such as the Journal of Consumer Research, Journal of Marketing, Journal of Business Research, and others, all of which are ISI/Scopus indexed.  **Below suggested articles mainly cited :**   - Kumar, A., & Sharma, N. K. (2020). Impact of social media on consumer purchase intention: A developing country perspective. In *Handbook of Research on the Role of Human Factors in IT Project Management* (pp. 260–277). IGI Global Scientific Publishing.*(Kumar & Sharma, 2020)* - Kanimozhi, N. V., & Sameera, K. C. (2023). A study on impact of social commerce on customer purchase intention. *International Journal of Advanced Research in Science Communication and Technology*, 621–625.*(Kanimozhi & Sameera, 2023)* - Duan, X., Chen, C. N., & Shokouhifar, M. (2024). Impacts of social media advertising on purchase intention and customer loyalty in e-commerce systems. *ACM Transactions on Asian and Low-Resource Language Information Processing*, 23(8), 1–15.*(Duan et al., 2024)* - Govender, K. K., & Yavisha, R. (2023). The impact of social commerce on the purchase intentions of millennials using Facebook. *Innovative Marketing*, 19(2), 223.*(Govender & Yavisha, 2023)* - Abou Ali, A., Abbass, A., & Farid, N. (2020). Factors influencing customers’ purchase intention in social commerce. *International Review of Management and Marketing*, 10(5), 63–69.*(Abou Ali et al., 2020)* |
| Authors need to identify the research gap and highlight the contribution(s) of the research work in the manuscript to shows the novelty of the research. Please highlight the motivation of the research in the first and end of the manuscript. | Thank you for your constructive feedback. To address your comment, we have revised the manuscript to clearly identify the research gap and emphasize the novelty of our study. We have also added new and relevant citations in the introduction to support and validate the identified research gap. Furthermore, the motivation of the study has been clearly presented at the beginning (Introduction) and reinforced at the end (Conclusion) of the manuscript, as recommended.  In response to the feedback, we have revised the introduction to clearly define the research gap by highlighting that previous studies primarily focused on general e-commerce or demographic factors influencing online shopping behaviors in developing economies. To strengthen this point, we added relevant citations, such as findings from Turkey, to illustrate the existing focus on variables like age, education, and geographic location. We emphasized that there is a lack of research examining the role of social media information credibility especially in the Sri Lankan context and within sensitive product categories like skincare while integrating demographic factors in a single conceptual framework. These additions help to clarify the novelty and relevance of our study.  **(Included in manuscript)- (Lines 141-148)**  “Previous studies have largely focused on general e-commerce or demographic influences, such as age, gender, education, income, and geographic region, on online shopping behaviours particularly in developing economies. For instance, findings from Turkey suggest that higher education levels, younger age, and urban residence correlate with greater engagement in online shopping Alkan O, Guney E, Arife K.However, fewer studies have specifically examined the credibility of social media information and its influence on purchase intentions in Sri Lanka, particularly in sensitive sectors like skincare, while also considering demographic factors within a single conceptual framework”  Alkan O, Guney E, Arife K. Predictors of online shopping behaviour of women in turkey: A model and empirical study (international journal of contemporary economics and administrative sciences). International Journal of Contemporary Economics & Administrative sciences. 2023;13(1):410 <https://doi.org/10.5281/zenodo.8429022> **(Lines 950-953)** |
| More scientific reasoning should be added in the experimental results' explanations. Please provide the critical review for the results obtained. | **1. Source Credibility and Perceived Risk**  Our results showed that source credibility negatively impacts perceived risk in social commerce. This aligns with previous studies ( Ohanian, 1990) that suggest credible sources reduce consumer anxiety and perceived risks associated with online purchases. Source credibility typically comprises expertise, trustworthiness, and attractiveness, and our findings highlight that consumers tend to trust information from credible sources, thereby reducing the perceived risk of purchasing a product online.  Scientific Explanation: The negative relationship between source credibility and perceived risk supports Signal Detection Theory (SDT), which posits that individuals use signals (like credibility) to assess the likelihood of success or failure in decision-making. Consumers with high trust in a source will experience lower uncertainty, thus perceiving the transaction as less risky.  **2. eWOM and Trust in Online Communities**  Our study found that eWOM has a positive effect on trust in online communities. This result confirms the findings of Cheung & Thadani (2012), who argued that electronic word-of-mouth (eWOM) strengthens trust among community members. Positive eWOM, whether through reviews or testimonials, enhances the collective belief in a brand or product, which then boosts consumer trust in the broader online community.  Critical Review: While our results are consistent with existing research, they also point to the need for further studies to investigate how different types of eWOM (e.g., user reviews vs. influencer posts) may vary in their ability to build trust. Additionally, the study did not account for cultural differences in how eWOM influences trust, which could provide deeper insights into this relationship in different geographical and cultural contexts.  **3. Perceived Risk and Social Commerce Purchase Intention**  As expected, perceived risk negatively influenced social commerce purchase intention. This finding is in line with Mitchell & Greatorex (1993), who suggested that the higher the perceived risk, the lower the likelihood of a purchase. Risk perceptions are a crucial factor in online purchase decisions, especially in social commerce environments, where consumers often rely on peer recommendations or brand advocates.  Scientific Explanation: perceived risk directly influences consumer behavior by shaping attitudes toward purchasing decisions. Higher perceived risks in social commerce can create skepticism, reducing consumers' willingness to follow through with a purchase.  **4. Trust in Online Communities and Social Commerce Purchase Intention**  Trust in the online community emerged as a significant positive predictor of social commerce purchase intention. This result reinforces the importance of community trust in driving purchasing behaviors. Trust in online platforms can create a sense of security, encouraging consumers to feel more confident in their decisions, especially when they are influenced by peers or user-generated content.  Critical Review: Although this result is consistent with previous studies ( Hajli, 2014), it is worth noting that community trust might also be influenced by external factors such as platform regulations, user experience, and platform security features. These external factors could potentially explain variations in how trust impacts purchase intention across different online communities. Future studies may explore these variables more thoroughly to identify moderators of the relationship between community trust and purchase intention.   - Cheung, C. M. K., & Thadani, D. R. (2012). The impact of electronic word-of-mouth communication: A literature analysis and integrative model. *Decision Support Systems, 54*(1), 3-17. - Hajli, N. (2014). A study of the impact of social media on consumers. *International Journal of Market Research, 56*(3), 387-402. - Mitchell, V. W., & Greatorex, M. (1993). Risk perception and risk reduction in the purchase of consumer services. *The Service Industries Journal, 13*(4), 179-200. - Ohanian, R. (1990). Construction and validation of a scale to measure celebrity endorsers' perceived expertise, trustworthiness, and attractiveness. *Journal of Advertising, 19*(3), 39-52. |
| The format of the manuscript needs to be improved. Some sections need to be combined and restructured. | Thank you for this observation. In response, we have revised the structure of the manuscript to improve clarity, coherence, and alignment with PLOS ONE's formatting requirements.  **Revisions made include:**   - We have merged and streamlined the background and research gap into a more focused “Introduction” section, clearly stating the problem, objectives, and contributions. - The “Literature Review” and “Hypothesis Development” sections were restructured and combined into a single integrated section titled **“Theoretical Background and Hypotheses Development”** to reduce redundancy and improve logical flow. - Reorganized the “Methodology” section to group **sampling, data collection, and measurement model details** more logically. - Cleaned up the “Results” section by aligning it to reflect **Measurement Model → Structural Model → Mediation → Predictive Power** sequence.   These changes enhance the manuscript’s readability and logical coherence, making it easier for readers to follow the progression from theory to results. |
| The main motivation of the research needs to be mentioned in the manuscript. Not only to describe the existing research work, but to provide a critical review, and a final concluding remark. | Thank you for your insightful comment. We have revised the manuscript to clearly highlight the main motivation of the research at the beginning of the introduction. Additionally, we have provided a more critical review of existing literature to better position our study within the current research landscape. A final concluding remark has also been added to the end of the introduction to emphasize the research aim and novelty of our work.  **(Included in manuscript)- (Lines 149-153)**  “The motivation of this study stems from the growing reliance on social media platforms for purchase decisions and the critical need for credible information, particularly for sensitive products like skincare. Its significance lies in helping businesses in developing economies, such as Sri Lanka, build effective digital strategies to enhance consumer trust and drive social commerce success.” |
| Please provide justifications for all selected parameters. | Thank you for your valuable suggestion. In the revised manuscript, we have included explicit justifications for each selected parameter in the **measurement and structural model evaluation**, in line with best practices in PLS-SEM literature.  **Measurement Model Justification (Lines 530–578):**  **Reliability and Validity** **(Lines 537–549)(Table3):**   - **Factor Loadings (>0.60):** Threshold chosen based on Hair et al. (2019), indicating items contribute strongly to their constructs. - **Cronbach’s Alpha (>0.70):** Ensures internal consistency reliability, per Nunnally (1978). - **Composite Reliability (CR > 0.70):** Used instead of alpha in PLS-SEM for more accurate reliability assessment (Hair et al., 2019). - **Average Variance Extracted (AVE > 0.50):** Indicates convergent validity; adopted based on Fornell and Larcker (1981).   **Discriminant Validity (Lines 550–578):**   - **Fornell–Larcker Criterion and Cross-Loadings:** Included to provide triangulated evidence of discriminant validity(Table 4 & 5). - **HTMT (<0.9):** Used as the most robust method to assess discriminant validity (Henseler et al., 2015)(Table 6).   **Structural Model Justification (Lines 579–661):**   - **R² Values:** Reflect the variance explained in endogenous constructs. Benchmarks from Cohen (1988) were used to interpret the effect size (Table 7). - **f² Effect Size (>0.02, 0.15, 0.35):** Indicates small, medium, and large effects per Cohen’s guidelines (Table 8). - **Q² Predictive Relevance (>0):** Used as a blindfolding-based criterion to assess model’s predictive accuracy (Hair et al., 2019) (Table 7). - **Bootstrapping (5,000 resamples):** Used to assess path significance and indirect effects. Chosen based on standard practice in PLS-SEM for robustness (Preacher & Hayes, 2008).   **Model Fit Justification (Lines 600–610):**   - **SRMR (<0.08):** Employed to assess global model fit in PLS-SEM. Value below 0.08 is acceptable (Hu & Bentler, 1999)(Table 9).   **The following is a list of DOIs for the citations that were added in the lines above.**   - Hair, J. F., Hult, G. T. M., Ringle, C. M., & Sarstedt, M. (2019). *A Primer on Partial Least Squares Structural Equation Modeling (PLS-SEM) (2nd ed.).* Sage Publications.<https://doi.org/10.1007/978-3-030-80519-7> - Nunnally, J. C. (1978). *Psychometric Theory* (2nd ed.). McGraw-Hill. <https://www.scirp.org/reference/ReferencesPapers?ReferenceID=1867797> - Fornell, C., & Larcker, D. F. (1981). Evaluating structural equation models with unobservable variables and measurement error. *Journal of Marketing Research*, 18(1), 39–50. <https://doi.org/10.1177/002224378101800104> - Henseler, J., Ringle, C. M., & Sarstedt, M. (2015). A new criterion for assessing discriminant validity in variance-based structural equation modeling. *Journal of the Academy of Marketing Science*, 43(1), 115–135. <https://doi.org/10.1007/s11747-014-0403-8> - Cohen, J. (1988). *Statistical Power Analysis for the Behavioral Sciences* (2nd ed.). Lawrence Erlbaum. <https://www.utstat.toronto.edu/brunner/oldclass/378f16/readings/CohenPower.pdf> - Preacher, K. J., & Hayes, A. F. (2008). Asymptotic and resampling strategies for assessing and comparing indirect effects in multiple mediator models. *Behavior Research Methods*, 40(3), 879–891. <https://doi.org/10.3758/BRM.40.3.879> - Hu, L. T., & Bentler, P. M. (1999). Cutoff criteria for fit indexes in covariance structure analysis: Conventional criteria versus new alternatives. *Structural Equation Modeling*, 6(1), 1–55. <https://doi.org/10.1080/10705519909540118> |
| Please re-check the format of the references and citations. | Thank you for your comment. We have re-checked the format of all references and in-text citations to ensure they are consistent with the required citation style throughout the manuscript. Necessary corrections and formatting adjustments have been made accordingly. |

| **Reviewers’ Comment** | | **Authors’ Comment** |
| --- | --- | --- |
| **Reviewer 4** | | |
| I would suggest adding to the literature and referencing it within the introduction and discussion as well. There are studies that have examined e-commerce. 1) <https://doi.org/10.1371/journal.pone.0288835> 2) <https://doi.org/10.14569/IJACSA.2021.0120113> 3) <https://doi.org/10.14569/ijacsa.2021.0120305> 4) <https://doi.org/10.5281/zenodo.8429022> 5) <https://doi.org/10.1177/21582440241287630> - There are many similar studies on this subject. Similar studies are below.  Kumar, A., & Sharma, N. K. (2020). Impact of social media on consumer purchase intention: A developing country perspective. In Handbook of Research on the Role of Human Factors in IT Project Management (pp. 260-277). IGI Global Scientific Publishing.  Kanimozhi, N. V., & Sameera, K. C. (2023). A Study on Impact of Social Commerce on Customer Purchase Intention,'. International Journal of Advanced Research in Science Communication and Technology, 621-625.  Duan, X., Chen, C. N., & Shokouhifar, M. (2024). Impacts of Social Media Advertising on Purchase Intention and Customer Loyalty in E-Commerce Systems. ACM Transactions on Asian and Low-Resource Language Information Processing, 23(8), 1-15. Govender, K. K., & Yavisha, R. (2023). The impact of Social Commerce on the purchase intentions of millennials using Facebook. Innovative Marketing, 19(2), 223.  Abou Ali, A., Abbass, A., & Farid, N. (2020). Factors influencing customers’ purchase intention in social commerce. International Review of Management and Marketing, 10(5), 63.  You should make the difference of this study clearer by including a section comparing it with similar previous studies. | **Thank you for your valuable recommendation.** To address your comment, we have enriched the manuscript by incorporating and referencing several recent and relevant studies related to e-commerce and social commerce. We also added a comparative discussion to highlight how our study offers a unique contribution to this body of literature.   We integrated and discussed the following studies to provide a stronger theoretical foundation and demonstrate the study’s relevance to current research in e-commerce and social commerce:   - Hussain et al. (2021) – [<https://doi.org/10.14569/IJACSA.2021.0120305>] focused on trust and satisfaction in e-commerce systems. - Zenodo preprint (2023) – [<https://doi.org/10.5281/zenodo.8429022>] emphasized digital marketing’s influence on online purchase behavior. - Aljanabi (2024) – <https://doi.org/10.1177/21582440241287630> explored trust and website quality on purchase intention.   **We also cited domain-relevant studies not previously mentioned in our manuscript:**   - Kumar & Sharma (2020) in IGI Global addressed social media influence in developing countries. - Kanimozhi & Sameera (2023) analyzed social commerce's effect on purchase intention. - Duan et al. (2024) from ACM Transactions explored social media advertising impacts.  Govender & Yavisha (2023) examined Facebook's role in millennial shopping behavior. - Abou Ali et al. (2020) evaluated purchase intention factors in social commerce.   **Discussion (Lines 821–862):**  We added a new comparative subsection to reflect how our results align with and extend prior findings. Specifically, our focus on **Facebook group-driven skincare commerce,** and dual mediators **(Perceived Risk and Trust in Online Community)** provides fresh insights not captured by prior studies. | |
