# Supplementary material for: Does social media information credibility influence social commerce purchase intention of skincare products? Evidence from Facebook
Source: PLoS One. 2025 Oct 22;20(10):e0334126. doi: 10.1371/journal.pone.0334126 (PMC12543200; doi:10.1371/journal.pone.0334126)
Supplement: Attachment — Submitted filename: Response to Reviewers R2.docx [file pone.0334126.s004.docx]

**Point-by-Point Response to the Reviewers**

Dear Reviewers,

We sincerely thank you for your thoughtful and constructive comments on our manuscript. We greatly appreciate the time and effort you have invested in reviewing our work. Your insights have been instrumental in helping us improve the quality and clarity of the paper. We have carefully considered each comment and have revised the manuscript accordingly. All responses and revisions are detailed point by point in the following document.

All the mentioned line numbers have been addressed and are clearly marked in the ***tracked version of the revised manuscript.***

| **Reviewers’ Comment** | **Authors’ Response** |
| --- | --- |
| **Reviewer 01** | |
| The authors have incorporated the suggested changes in the abstract, introduction, literature review, methodology, results, discussion, and conclusion. Therefore, the paper could now be accepted for publication. | Thank you very much for your thoughtful and constructive feedback throughout the review process.  We sincerely appreciate your positive evaluation and are grateful that you have acknowledged the revisions made to the abstract, introduction, literature review, methodology, results, discussion, and conclusion. Your insights were instrumental in enhancing the clarity and overall quality of our manuscript.  We are pleased to know that the paper is now considered suitable for publication, and we truly value your recommendation. |
|  | |
| **Reviewers’ Comment** | **Authors’ Response** |
| **Reviewer 05** | |
| 01. Introduction: The introduction section does not properly reveal the research gap. What is the novelty of this proposed research in terms of theory and managerial contributions? Major work is required to justify this research's novelty and prospective implications.  02. What is the current research progress on the topic? For example, what has been explored before, and why are you pursuing this area?    03. What are the theoretical gaps? (e.g., scholarly evidence of contradictory findings and underexplored relationships) and why they should be addressed (e.g., why addressing them is necessary, important, useful, and urgent?)  04. What are the practical gaps? (e.g., market and news reports of underlying problems and untapped opportunities) and why they should be addressed (e.g., why addressing them is necessary, important, useful, and urgent?);  05. What are the expected contributions to theory? (e.g., what new understanding can be derived?); and  06. What are the expected contributions to practice? (e.g., what real-world issues can be solved?).  07. The authors proposed the research about skin care industry. There must be a brief Sri Lankan skin care industry background, particularly in the context of social commerce. Source credibility of what either consumers or firms?  08. Although the authors discussed UGC in one or two sentences. It’s not enough to justify your arguments. The authors must provide the justifications as a lot of research has been done on the proposed constructs in the context of social media.  09. The authors generally discussed social media users. How many people use Facebook in Sri Lanka? Social media facts and figures must be related to Sri Lanka not in general terms. | Thank you for highlighting the need to better reveal the research gap and clarify the novelty of our study.  We have highlighted the research gap in our first revision. As per your comment, authors have further elaborated on the research gap and contribution in revised version. Therefore, we have revised the introduction section to strengthen the research gap.  We believed that the revised manuscript now clearly explains the research gap and theory behind the study. It shows that most past studies on online shopping focused on general e-commerce and demographics, with little attention to credibility factors.  Then, it also points out that many of these studies were done in developed countries and did not explore how source credibility, e-WOM, trust, and perceived risk work together especially for trusted products like skincare.  This study is new because it looks at social commerce in Sri Lanka, an emerging market. It combines credibility factors in one framework and considers how demographics may affect results. Studying a trust-based product like skincare makes the findings more useful for businesses.  Overall, the updated introduction and theory section clearly shows why this study is original and important.  Included in revised manuscript **(Line numbers 164-190)**  *“Previous research on online shopping behaviour has predominantly focused on general e-commerce or demographic factors such as age, gender, education, income, and geographic location (**27**,* *28**). For instance, a study in Turkey found that younger consumers with higher education and urban residence were more inclined to engage in online purchases (**29**). Other studies have explored the effects of either source credibility or electronic word-of-mouth (e-WOM) credibility in isolation on consumer trust or purchase decisions (**24**,* *30**). However, there is limited research that integrates these credibility dimensions within a unified framework, particularly alongside trust and perceived risk, to explain purchase intention in social commerce.”*  *“While social commerce literature has advanced in exploring individual factors like trust, engagement, and platform usability, most research has focused on general products like electronics, fashion, and services (**31**). These studies are also largely conducted in developed economies, limiting their applicability to emerging markets. Little attention has been given to how credibility-related factors interact in credibility-sensitive sectors like skincare, where purchase decisions are strongly influenced by trust due to personal and health-related implications”.*  *“Although skincare brands increasingly use social media to influence purchase behaviour. existing studies rarely assess how consumers evaluate the credibility of this content when making purchase decisions particularly for products that require a high level of consumer trust (**19**). In addition, research in developing economies such as Sri Lanka where social media usage is rising but trust in digital content remains uncertain is still limited”.*  *“This study addresses these gaps by proposing an integrated model that examines the influence of source credibility and e-WOM credibility on purchase intention, mediated by trust in online communities and perceived risk, specifically within the skincare industry in Sri Lanka, while also incorporating demographic variables as contextual factors. Therefore, it contributes both theoretical insight and practical guidance for businesses operating in credibility-sensitive markets within emerging economies.”*  Newly added citations   - Vithana L.V.M.G, Fernando P.I.N. Impact of e-word of mouth (e-wom) on consumers’ purchase intention for cosmetic products: The mediating role of brand image (with reference to facebook users). Sri Lanka Journal of Marketing. 2024;10(1):150-69. <https://doi.org/10.4038/sljmuok.v10i1.171> - R.A.S.D. Rathnayake, Lakshika V.G.P. Impact of social media influencers’ credibility on the purchase intention: Reference to the beauty industry. Asian Journal of Marketing Management. 2023;2(1):103-37. <https://doi.org/10.31357/ajmm.v2i01.6254> - Erkan I, Evans C. The influence of ewom in social media on consumers’ purchase intentions: An extended approach to information adoption. Computers in Human Behavior. 2016;61(2):47-55. <https://doi.org/10.1016/j.chb.2016.03.003> - Filieri R, McLeay F. E-wom and accommodation. Journal of Travel Research. 2013;53(1):44-57. <https://doi.org/10.1177/0047287513481274> - Akar E, and Topçu B. An examination of the factors influencing consumers' attitudes toward social media marketing. Journal of Internet Commerce. 2011;10(1):35-67. <https://doi.org/10.1080/15332861.2011.558456> - Alkan O, Guney E, Arife K. Predictors of online shopping behavior of women in turkey: A model and empirical study (international journal of contemporary economics and administrative sciences). International Journal of Contemporary Economics & Administrative sciences. 2023;13(1):410-42. <https://doi.org/10.5281/zenodo.8429022> - Chen Z, Cao H, Xu F, Cheng M, Wang T, Li Y. Understanding the role of intermediaries in online social e-commerce: An exploratory study of beidian. Proceedings of the ACM on Human-Computer Interaction. 2020;4:1-24. <https://doi.org/10.1145/3415185>   Thank you for bringing out this important point about the current research and the need for our study.  Primarily, in the revised manuscript, we have expanded the literature review and introduction to better explain the current level of knowledge in the area and what we are contributing to this domain.  While previous studies have looked at source credibility, e-WOM, trust, and perceived risk in online shopping, they have often studied these factors separately and mainly in general product areas like electronics and fashion. Also, most research focuses on developed countries, with little attention to how these factors work together in credibility-sensitive products like skincare, especially in emerging markets.  Authors have identified the lack of understanding about how consumers in developing countries assess credibility cues when buying high-trust products. Skincare is a unique and underexplored area where trust is crucial due to its personal and health related nature.  Therefore, to fill this gap, we propose a framework that combines source and e-WOM credibility, with trust in online communities and perceived risk as mediators, to explain purchase intention in Sri Lanka’s skincare market. This not only adds to social commerce theory but also offers useful insights for businesses.  Included in revised manuscript **(Line numbers 164-179)**  In the research gap highlighted the current research progress on the topic  *“While social commerce literature has advanced in exploring individual factors like trust, engagement, and platform usability, most research has focused on general products like electronics, fashion, and services (**31**). These studies are also largely conducted in developed economies, limiting their applicability to emerging markets. Little attention has been given to how credibility-related factors interact in credibility-sensitive sectors like skincare, where purchase decisions are strongly influenced by trust due to personal and health-related implications.”*  *“This study addresses these gaps by proposing an integrated model that examines the influence of source credibility and e-WOM credibility on purchase intention, mediated by trust in online communities and perceived risk, specifically within the skincare industry in Sri Lanka, while also incorporating demographic variables as contextual factors. Therefore, it contributes both theoretical insight and practical guidance for businesses operating in credibility-sensitive markets within emerging economies.”*  *Sri Lanka’s skincare industry has grown in recent years due to better health awareness, urban living, and more social media use among young people. Brands use platforms like Facebook and Instagram to reach customers through influencers and user content. However, weak rules and false product claims have raised trust issues. This makes the skincare market in Sri Lanka a useful area to study trust and credibility in social commerce.*  Included in manuscript **(Line numbers 132-140)**  *“Sri Lanka’s skincare industry has shown consistent growth in recent years, fuelled by rising health consciousness, urbanization, and increased social media usage among younger demographics (**18**). Platforms such as Facebook and Instagram have become vital channels for skincare brands to engage consumers through influencer campaigns, user-generated content, and direct-to-consumer promotions (**18**,* *19**). However, the industry faces credibility challenges due to limited regulatory oversight and the proliferation of unverified product claims online (**20**). These dynamics make the skincare sector in Sri Lanka a critical context for examining trust and information credibility in social commerce (**18**,* *20**,* *21**).*  Thank you to the reviewer for pointing out the lack of clear theoretical gaps.  Primarily, in the revised manuscript, we have clearly explained the key gaps and our reasons for addressing them.  In response, we have strengthened the manuscript by clearly explaining the theoretical contributions of our study. We address the gap in past research where source credibility, e-WOM credibility, trust, and perceived risk were often studied separately. Then, our study presents an integrated framework showing how these factors together influence purchase intention in social commerce.  Hence, we also clarify the unclear relationship between trust and risk by testing their mediating roles. The framework is applied to the skincare industry a credibility-sensitive and underexplored product category and focused on Sri Lanka, an emerging market with growing social media use but limited research on digital trust. Hence, these additions provide a deeper understanding of credibility based consumer behavior and are now reflected in the revised manuscript.  Included in revised manuscript **Line numbers 228-234)**  *“Despite the expanding literature on social commerce, most studies examine source credibility, e-WOM, trust, and perceived risk as discrete constructs, lacking a unified framework that captures their combined impact on consumer purchase intention particularly in high-involvement categories like skincare, where decisions are highly trust-dependent (**18**). Additionally, the trust risk relationship remains theoretically ambiguous, with prior research reporting contradictory mediating roles and causal directions (**32**,* *33**). It limits clarity on how credibility perceptions operate in sensitive decision-making contexts”.*  Newly added citations   - Vithana L.V.M.G, Fernando P.I.N. Impact of e-word of mouth (e-wom) on consumers’ purchase intention for cosmetic products: The mediating role of brand image (with reference to facebook users). Sri Lanka Journal of Marketing. 2024;10(1):150-69. <https://doi.org/10.4038/sljmuok.v10i1.171> - Pavlou P.A. Consumer acceptance of electronic commerce: Integrating trust and risk with the technology acceptance model. International Journal of Electronic Commerce. 2003;7(3):101-34. <https://doi.org/10.1080/10864415.2003.11044275>   Thank you for bringing out this important point about the lack of practical gaps.  We have expanded the revised manuscript to explain the practical importance of our study.  Therefore, while skincare brands increasingly use social media to engage consumers, concerns are growing especially in markets like Sri Lanka about the credibility of digital content, influencer promotions, and unverified product claims. Then, weak regulations and low consumer awareness make it harder for consumers to trust online shopping.  Our study addresses this by showing how source and e-WOM credibility influence trust and perceived risk, offering practical guidance for marketers. This helps businesses create more trustworthy communication and protect consumers in high-trust product markets like skincare.  Therefore, given the rapid growth of social commerce in loosely regulated digital spaces, we believe these insights are both timely and important  Included in revised manuscript  **(Line numbers (235-241)**  “Practically, while consumers increasingly rely on social media for product evaluation, this trust is undermined especially in developing markets like Sri Lanka, where digital adoption coexists with content skepticism. *Reports* cite growing concerns over influencer credibility, fake reviews, and unverifiable claims, which reduce conversion and weaken brand loyalty in sectors like skincare (34, 35). Addressing these gaps is urgent thus theoretically, it advances credibility-based models in social commerce. Therefore, practically, it informs trust-driven strategies to improve marketing effectiveness in emerging markets.”  Thank you for bringing out this important point about the expected contribution to theory.  Accordingly, in the revised manuscript, we clearly explain these contributions. While past research treated source credibility, e-WOM, trust, and perceived risk separately, our study combines them into one framework to show how they together affect purchase decisions, especially for trust-sensitive products like skincare. Hence, this approach gives a clear understanding of how credibility affects consumer behavior in social commerce.  Accordingly, we also clarify the unclear relationship between trust and perceived risk, which past studies have shown in different and conflicting ways. By testing these in our study, we help clear up these inconsistencies and explain how trust and risk work together in consumer decisions. Therefore, these points are now included in the revised manuscript  Included in revised manuscript (**Line numbers 228-234)**  *“Despite the expanding literature on social commerce, most studies examine source credibility, e-WOM, trust, and perceived risk as discrete constructs, lacking a unified framework that captures their combined impact on consumer purchase intention particularly in high-involvement categories like skincare, where decisions are highly trust-dependent (**18**). Additionally, the trust risk relationship remains theoretically ambiguous, with prior research reporting contradictory mediating roles and causal directions (**32**,* *33**). It limits clarity on how credibility perceptions operate in sensitive decision-making contexts”.*  Thank you for bringing out this important point about the expected contribution to practice.  In the revised manuscript, we explain how our study helps real-world practice. Therefore, our findings assist marketers and brand managers in the skincare industry to understand how source credibility and e-WOM credibility affect consumer trust and perceived risk, which are important for purchase decisions in social commerce. This is especially important for skincare, where consumers need extra assurance due to personal and health concerns.  Therefore, the study also gives useful insights for businesses in emerging markets like Sri Lanka, where digital marketing rules are weak and consumer trust is fragile. By showing which credibility factors most affect trust and buying decisions, our research helps create more honest, clear, and effective marketing strategies.  Included in revised manuscript **(Line numbers 235-241)**  *“Practically, while consumers increasingly rely on social media for product evaluation, this trust is undermined especially in developing markets like Sri Lanka, where digital adoption coexists with content skepticism. Reports cite growing concerns over influencer credibility, fake reviews, and unverifiable claims, which reduce conversion and weaken brand loyalty in sectors like skincare (**34**,* *35**). Addressing these gaps is urgent thus theoretically, it advances credibility-based models in social commerce. Therefore, it informs trust-driven strategies to improve marketing effectiveness in emerging markets.”*  Thank you for bringing out this important point about the need to include background on the Sri Lankan skincare industry.  In response, we added a new paragraph to the revised manuscript that gives a brief overview of Sri Lanka’s skincare industry, especially in social commerce. Hence, this section explains the recent growth due to rising health awareness and social media use, particularly among younger people. We also highlight the increasing use of platforms like Facebook and Instagram for influencer marketing, user content, and direct customer engagement.  Therefore, we have also clarified the definition of source credibility as used in our study. It includes both firms or influencers such as sponsored posts or brand messages and peer consumers like user reviews or recommendations on social media. This is now clearly explained in the revised manuscript.  We added this background section to make the study clear and better grounded. These changes help explain why the Sri Lankan skincare market is a essential place to study credibility and trust in social commerce.  Included in revised manuscript **(Line numbers 132-140)**  *“Sri Lanka’s skincare industry has shown consistent growth in recent years, fuelled by rising health consciousness, urbanization, and increased social media usage among younger demographics (**18**). Social commerce platforms such as Facebook and Instagram have become vital channels for skincare brands to engage consumers through influencer campaigns, user-generated content, and direct-to-consumer promotions (**18**,* *19**). However, the industry faces credibility challenges due to limited regulatory oversight and the proliferation of unverified product claims online (**20**). These dynamics make the skincare sector in Sri Lanka a critical context for examining trust and information credibility in social commerce (**18**,* *20**,* *21**).”*  Newly added citations   - Vithana L.V.M.G, Fernando P.I.N. Impact of e-word of mouth (e-wom) on consumers’ purchase intention for cosmetic products: The mediating role of brand image (with reference to facebook users). Sri Lanka Journal of Marketing. 2024;10(1):150-69. <https://doi.org/10.4038/sljmuok.v10i1.171> - R.A.S.D. Rathnayake, Lakshika V.G.P. Impact of social media influencers’ credibility on the purchase intention: Reference to the beauty industry. Asian Journal of Marketing Management. 2023;2(1):103-37. <https://doi.org/10.31357/ajmm.v2i01.6254> - FT D. Sri lanka’s beauty industry urged to build trust through transparency 2024 [Available from: <https://www.ft.lk/> - International E. Beauty and personal care in sri lanka. [Online Statistical Report]. 2024 [Available from: <https://www.euromonitor.com/>.   Thank you for bringing out this important point about the need to further justify our use of user-generated content (UGC).  Authors have expanded the discussion of UGC in the revised manuscript to better show its importance in social commerce. The new paragraph explains that UGC like reviews, ratings, testimonials, and social media comments plays a key role in shaping how consumers see trust and credibility. Therefore, we also point out that while UGC is often studied, its role in trust-sensitive areas like skincare and in emerging markets like Sri Lanka is still not well understood. Additionally, we include recent data showing that consumers often doubt the credibility of UGC, but it usually has more impact on buying decisions than content from companies. Hence, these changes make UGC a clear and important part of our study and show how it affects consumer choices online.  Included in revised manuscript **(Line numbers 145-159)**  *“User-generated content (UGC) such as reviews, ratings, testimonials, and social media comments plays a central role in shaping consumer perceptions of credibility and trust in social commerce environments (**22**). Consumers often question the trustworthiness of user-generated content, such as reviews, ratings, and recommendations. Many are concerned that information on social media could be biased, exaggerated, or even false, leading to doubts about its reliability (**19**,* *22**). Research shows that 70% of online consumers worry about the credibility of information they find on social media platforms (**23**). Prior research confirms that UGC significantly influences consumers' attitudes, risk perceptions, and purchase intentions, often more than firm-generated content (**24**,* *25**). User generated content serves as a form of electronic word-of-mouth (e-WOM), where consumers perceive peer opinions as more authentic and trustworthy (**26**). Despite extensive literature on user generated content, its impact within credibility sensitive industries like skincare, and in understudied digital markets such as Sri Lanka, remains underexplored.”*  Newly added citations   - R.A.S.D. Rathnayake, Lakshika V.G.P. Impact of social media influencers’ credibility on the purchase intention: Reference to the beauty industry. Asian Journal of Marketing Management. 2023;2(1):103-37. <https://doi.org/10.31357/ajmm.v2i01.6254> - Erkan I, Evans C. The influence of ewom in social media on consumers’ purchase intentions: An extended approach to information adoption. Computers in Human Behavior. 2016;61(2):47-55. <https://doi.org/10.1016/j.chb.2016.03.003> - Filieri R, McLeay F. E-wom and accommodation. Journal of Travel Research. 2013;53(1):44-57. <https://doi.org/10.1177/0047287513481274>   Thank you for bringing out this important point about the need to include specific social media usage data for Sri Lanka.  We have updated the manuscript with recent statistics showing that, in 2024, Facebook is the top social media platform in Sri Lanka with about 7.45 million active users, representing over 34% of the population. Instagram follows with approximately 2.5 million active users, while TikTok has around 1.8 million users in the country. This local data highlights the importance of focusing on social commerce in Sri Lanka and shows the strong influence of these platforms especially Facebook on consumer behavior.  Included in revised manuscript **(Line number 124-126)**  *“With 3.2 billion active users worldwide, Facebook also remains the leading platform in Sri Lanka, with approximately 7.45 million active users as of 2024 accounting for over 34% of the national population (**15**)”.*  Newly added citations   - DataReportal. Digital 2024: Sri lanka [Online Statistical Report]. DataReportal 2024 [Available from: <https://datareportal.com/> |
| Literature review: First paragraph is irrelevant, must be removed. Instead, the authors should focus only on the proposed constructs. For Source Credibility, the authors already explained that “Balouchi, Aziz (34), has demonstrated a significant relationship between source credibility and perceived risk in the context of online purchasing.” If this relationship is already documented in the literature, what is the novelty of this article? Similarly, eWOM Credibility (see page 11) “communication information technology have significantly boosted e WOM elements, including reviews, comments, and recommendations, and positively impacted consumers' trust, which certainly strengthens their purchase intentions (6).” The authors must find the gap in the literature to justify this publication. | Thank you for this insightful comment. As you have suggested, we have removed the initial paragraph of the literature review to improve focus and relevance. The revised section now directly discusses the proposed constructs. (**Line Numbers 288 -294, 305 -325**) Regarding source credibility and e WOM credibility, while previous studies have identified their direct effects on perceived risk and trust, our study contributes by addressing how these effects occur through mediation and sequential mediation (i.e., via trust and perceived risk) within a social commerce context using the Elaboration Likelihood Model (ELM) as the guiding framework. This integration has not been sufficiently explored in existing literature, particularly in the context of emerging markets like Sri Lanka.  While previous studies have shown that source credibility and e WOM credibility can directly influence trust and reduce perceived risk, our study adds new value by going a step further. We examine how these effects happen specifically through the mediating roles of trust and perceived risk, and also through a sequential . This approach is guided by the Elaboration Likelihood Model (ELM), Petty R, Cacioppo J. The elaboration likelihood model of persuasion. *Advances in Experimental Social Psychology*. 1986;19:123-205. <https://doi.org/10.1016/S0065-2601(08)60214-2>  which explains how people process information and make decisions based on both the quality of the message and the credibility of the source.  What makes our study different is that we apply this model in a real social commerce setting, focusing on skincare consumers in Sri Lanka who purchase through Facebook. This group is important because social commerce in emerging markets is growing fast but is still under-researched. Most past studies were done in developed countries or on general e-commerce users. By focusing on a specific product category (skincare), a specific platform (Facebook), and a specific market (Sri Lanka), we fill a gap in the literature and provide new insights into how online trust and risk affect purchase behavior in real-life settings. We have emphasized this gap and our study's theoretical novelty in both the literature review and discussion sections. |
| The proposed model shows mediator only Perceived instead of Perceived Risk. I think it needs to adjust the font to highlight the complete name of the variable. Which theories support this framework and how? There must be a theory section which supports this framework and how the proposed theories support this framework. The theory support is weak and quite unsatisfactory in the context of hypothesis development. The hypotheses must be developed in theory contextualization. It requires major rewriting. | Thank you for your constructive feedback. As advised, we have corrected the labeling in the conceptual model to display the complete term “Perceived Risk” for clarity, ***Fig 2. Conceptual Framework.***  (**Line Numbers 469 -520**) In addition, we have substantially revised the theoretical foundation of the manuscript by explicitly incorporating the **Elaboration Likelihood Model (ELM) by** **Petty & Cacioppo (1986)** as the guiding theory. The updated section explains how consumers process online information through two routes: the **central route**, which involves thoughtful evaluation of message content (e.g., e WOM credibility), and the **peripheral route**, which relies on surface cues such as source credibility. We applied this model to explain how **trust in the online community** and **perceived risk** act as key cognitive factors that influence purchase intention in a social commerce environment.  To strengthen the theoretical alignment, we also rewrote the **hypotheses development section** so that each hypothesis is now clearly supported by the ELM. For example, **H_1_** is based on the idea that a credible source can reduce uncertainty (a peripheral cue), leading to a lower perceived risk. **H_2_** reflects central-route processing, where consumers build trust when they perceive e WOM content to be reliable and relevant. **H_3_–H_5_** are grounded in the ELM’s explanation of how lower perceived risk and higher trust increase the likelihood of behavioral intention. Finally, **H_6_–H_8_** test the mediating and sequential roles of trust and risk, showing how credibility influences purchase behavior through these deeper psychological pathways. These changes directly address your comment and strengthen both the theoretical foundation and the logical flow of the proposed model.  Petty R, Cacioppo J. The elaboration likelihood model of persuasion. *Advances in Experimental Social Psychology*. 1986;19:123-205. <https://doi.org/10.1016/S0065-2601(08)60214-2> |
| Research design too complex and added unnecessary details. For example, positivism philosophy approach and deductive approach. What is the difference between them. Since the model is too simple, I don’t think such philosophy should be used. It’s a simple quantitative research study. What was the sampling approach? For data collection, which social media platforms were used? How did you identify the targeted population? | Thank you for your valuable feedback.  **(Line Numbers 549 -563**) We have simplified the research design section by removing philosophical explanations and focusing only on the essential methodological steps. We now clearly state that the study followed a **quantitative research approach**, using a **survey method** to collect data and **statistical analysis** to test the hypotheses. These changes make the section more concise and aligned with the straightforward nature of our model and research objectives. Additionally,  (**Line Numbers 562 – 577**) We have clearly stated that a **convenience sampling method** was used to collect data from **Facebook users in Sri Lanka** who have **prior experience purchasing skincare products through social commerce** (Statista, 2024). To ensure that the responses were relevant to the study, we included **screening questions** at the beginning of the survey to confirm that participants had engaged in such purchases. This helped us accurately identify the **target population**, focusing only on users who are familiar with using Facebook for online shopping, specifically for skincare products.  Statista. Biggest social media platform by users [Online Statistical Report]. Statista; 2024 [cited 2024 December 20]. Available from: <https://www.statista.com/statistics/272014/global-social-networks-ranked-by-number-of-users/>. |
| The authors mainly collected data from users (282) who used Facebook Once a month or less. This creates a serious doubt about the credibility of the findings. Since Facebook is a social media platform where users use it for fun while online purchasing is too complex and users frequently use online platforms to ensure accurate online shopping. Proper justification required. | Thank you for your thoughtful and important comment. We would like to respectfully clarify that the total sample size of our study is **384 respondents**, not 282. It appears there may have been a misunderstanding, possibly based on a misreading of the descriptive statistics. More importantly, we applied **screening criteria** at the beginning of the survey to include only **Facebook users who have experience in purchasing skincare products through the platform** or those who actively engage with social commerce content such as reviews, comments, and product inquiries. Respondents who indicated minimal or no engagement in Facebook-based purchasing were excluded from the final analysis.  We also acknowledge the concern regarding Facebook’s general use as an entertainment platform. However, in **Sri Lanka**, Facebook is not only the most widely used social media platform (Statista, 2024), but it is also commonly used for **social commerce**, particularly for skincare and fashion-related products. Many small businesses and individual sellers in Sri Lanka rely on Facebook pages and groups for direct product promotion and customer interaction. Given this reality, Facebook was a highly relevant and suitable platform for data collection in our study.  Finally, our focus on **skincare product buyers** via Facebook helps us explore **a specific and active consumer segment** within the growing field of social commerce, an area that remains underrepresented in academic literature, especially in the context of **emerging markets**. We have clarified these points in the methodology section to strengthen the credibility and contextual appropriateness of our research. |
| Table 3 should include the items used for data collection. | Thank you for your helpful suggestion. In the revised manuscript, we have updated Table 3 to include the actual survey items used for each construct to improve clarity and transparency of the measurement instrument.  Included in revised manuscript **(page numbers 28-31)** |
| The discussion part is weak. It is unclear which results contribute to which theoretical stream and how you position your study's results. | Thank you for your valuable feedback. (**Line Numbers 786 – 885**) We have thoroughly revised the discussion section to explicitly connect each key finding to the relevant theoretical framework, with a particular focus on the Elaboration Likelihood Model (ELM) (Petty & Cacioppo, 1986). “Specifically, we clarify how our results support the dual-route processing concept of ELM: source credibility aligns with the peripheral route, influencing consumers through cues related to the source’s trustworthiness, while e WOM credibility corresponds to the central route, where consumers engage in more thoughtful evaluation of message content. We further explain how trust and perceived risk serve as important mediators within this model, illustrating the cognitive mechanisms by which information credibility impacts social commerce purchase intentions”.  Moreover, we situate our findings within the broader body of existing literature, emphasizing how our study contributes to advancing understanding of consumer decision-making in social commerce settings, particularly in emerging markets like Sri Lanka, which have been underrepresented in prior research. These revisions enhance both the theoretical clarity and practical relevance of our study, providing valuable insights for academics and practitioners seeking to better understand and leverage information credibility factors in online consumer behavior.  Petty R, Cacioppo J. The elaboration likelihood model of persuasion. *Advances in Experimental Social Psychology*. 1986;19:123-205. <https://doi.org/10.1016/S0065-2601(08)60214-2> |
| Theoretical implications can be improved. For example, what has been done before? What are the new contributions and their significance in literature? | Thank you for this suggestion. We appreciate your input and have taken it into careful consideration during our revisions. (**Line Numbers 88 – 915**) We have enhanced the theoretical implications by clearly outlining the relevant existing literature and identifying specific gaps that our study aims to address. While previous research has examined the individual effects of source credibility and e WOM credibility on consumer behavior, limited attention has been paid to how these factors influence purchase intention through mediating mechanisms such as trust and perceived risk, particularly within the context of social commerce in emerging markets.  By integrating the Elaboration Likelihood Model (ELM) into our framework, we offer a new perspective on the dual processing routes (central and peripheral) through which consumers evaluate information in online environments. Specifically, we demonstrate how source credibility functions as a peripheral cue and e WOM credibility represents a central message-based cue, both influencing purchase intention via psychological mediators. This theoretical application of ELM provides deeper insight into the cognitive and emotional pathways involved in consumer decision-making in social commerce settings.  Furthermore, our study contributes to the literature by applying this model in the context of Facebook-based skincare product purchases in Sri Lanka, a highly relevant yet underexplored setting. These findings not only extend the applicability of ELM to social commerce research but also respond to calls for more empirical studies focused on user behavior in emerging economies, where trust and perceived risk play critical roles in shaping online purchase behavior. (Erkan & Evans, 2016), to help them build trust and reduce perceived risk among social commerce consumers.  Erkan I, Evans C. The influence of ewom in social media on consumers’ purchase intentions: An extended approach to information adoption. Computers in Human Behavior. 2016;61(2):47-55. <https://doi.org/10.1016/j.chb.2016.03.003> |
| The managerial contributions can be more emphasized and fine-tuned, given the study context. I would suggest using some literature support in this part as well. | Thank you for your valuable suggestion. (**Line Numbers 917 – 948**) We have revised the managerial contributions section to provide more focused and actionable insights specifically tailored to the social commerce context in emerging markets like Sri Lanka. Recognizing the unique challenges faced in these markets, such as higher perceived risks and varying levels of trust in online platforms, we emphasized strategies that marketers and platform managers can adopt to build stronger consumer confidence.  To strengthen this section, we incorporated relevant literature that underscores the importance of **building trust** through transparent communication, credible information sources, and fostering active user engagement (Gefen, 2000; Pavlou, 2003). Additionally, managing **perceived risk** by providing clear product information, facilitating customer reviews, and ensuring secure transaction processes can significantly reduce consumers' hesitations and encourage purchase behavior.  These additions not only provide a stronger theoretical foundation for our recommendations but also enhance their practical relevance. By applying these evidence-based approaches, marketers and social commerce platform managers can effectively increase consumer trust, reduce perceived risks, and ultimately drive higher purchase intentions, especially within emerging market contexts where online shopping behaviors are still developing.  Gefen D, Karahanna E, Straub DW. Trust and tam in online shopping: An integrated model. MIS Quarterly. 2003;27(1):51-90. <http://www.jstor.org/stable/30036519>  Pavlou PA. Consumer acceptance of electronic commerce: Integrating trust and risk with the technology acceptance model. International Journal of Electronic Commerce. 2003;7(3):101-34. <https://doi.org/10.1080/10864415.2003.11044275> |
| The quality of communication is average. You need to proofread the whole article to simplify and use concise language and improve the language expression of the article. | Thank you for your valuable feedback. We carefully checked the entire manuscript for grammar, language clarity, and expression. The article has been thoroughly proofread to ensure concise and clear communication throughout. |
