# Supplementary material for: Does social media information credibility influence social commerce purchase intention of skincare products? Evidence from Facebook
Source: PLoS One. 2025 Oct 22;20(10):e0334126. doi: 10.1371/journal.pone.0334126 (PMC12543200; doi:10.1371/journal.pone.0334126)
Supplement: Attachment — Submitted filename: Response to Reveiwers.docx [file pone.0334126.s005.docx]

**Point-by-Point Response to the Reviewers**

Dear Reviewers,

We sincerely thank you for your thoughtful and constructive comments on our manuscript. We greatly appreciate the time and effort you have invested in reviewing our work. Your insights have been instrumental in helping us improve the quality and clarity of the paper. We have carefully considered each comment and have revised the manuscript accordingly. All responses and revisions are detailed point by point in the following document.

| **Reviewers’ Comment** | **Authors’ Response** |
| --- | --- |
| Terms such as “social commerce purchase intention,” “e-WOM,” and “information credibility” should be used uniformly across abstract, methods, and discussion. Minor inconsistencies remain. | Thank you for your valuable and insightful feedback. We have thoroughly reviewed the manuscript and standardized the terminology throughout. Now we used consistently across the abstract, methodology, and discussion sections to avoid any confusion. |
| Please report exact p-values and effect sizes where applicable, instead of only threshold reporting (e.g., “p < 0.05”). This will enhance statistical transparency. | Thank you for this valuable suggestion. We have revised the Results section and related tables to report exact p-values rather than threshold reporting. |
| Figure legends should be more self-contained, explicitly including:   - sample size (n) - type of statistical test applied - meaning of significance indicators (*, **, etc.). | We appreciate this suggestion. The figure legends have been revised to include the sample size (n), statistical test applied, and explanations for significance indicators. |
| While expanded, the discussion still leans towards summarizing findings. I recommend:   - More critical engagement with contrasting studies, not just supportive literature. - A clearer acknowledgment of limitations (e.g., convenience sampling, generalizability to broader populations, reliance on self-reported data). | We sincerely thank the reviewer for this valuable feedback, which helped us strengthen the discussion and refine the overall contribution of the paper. As recommended **(Line 818-833)**, we have expanded the discussion section to include more critical engagement with contrasting studies. Specifically, we now acknowledge that while our findings support the positive effects of source credibility and eWOM credibility, some prior studies highlight the potential for conflicting outcomes, such as when influencer motives are questioned or when excessive user-generated content leads to information overload. This addition ensures that our discussion is not only grounded in supportive literature but also critically considers opposing perspectives, thereby providing a more balanced interpretation of the results.  *“Nonetheless, it is worth noting that some previous studies do not corroborate the outcomes of this research (58). Some experts believe that the concept of source credibility does not entail a lower perceived risk, nor even a lower risk perception, if there are suspicions that the endorser of an influencer marketing campaign is driven by profit, or if there is an overabundance of marketing promotions (**38**,* *41**). In the same manner, the studies that are more on the pessimistic side of the spectrum assert that e WOM is positively correlated with trust, an emphasis that is needed because there is an overabundance of user-generated reviews that can, at times, lead to a state of confusion, scepticism, or even an overage of information, ultimately decreasing the trust of the consumer (**6**). In some instances, due to a more formal reliance on certificates and sophisticated control mechanisms, the so-called peer-generated credibility may be of a lower order (**17**,* *42**). The differences outlined above affirm that the effects of source credibility and e WOM credibility are not universal; they are influenced by attitude, the general level of consumer education, and the prevailing industry standards (**52**,* *53**). As a result, the new findings outlined above ought to be treated more delicately; particularly, they should be treated with the understanding that the factors of source credibility will not always mitigate risk and increase trust in all situations concerning social commerce (2)”.*  In addition, **(Line 952-955)**, we have addressed the reviewer’s suggestion regarding the limitations of our study. We have clearly acknowledged that the use of convenience sampling, reliance on self-reported data, and the Sri Lankan Facebook user context limit the generalizability of our findings to broader populations and other platforms. This expanded acknowledgment highlights the boundaries of our research and underscores the need for caution in interpreting the findings beyond the studied context.  *“As a result, the conclusions might not be true for users of other social media  ecosystems. The accuracy and response reliability, bias, and omissions to self-reported metrics are issues addressed in this study (**17**,* *42**). Furthermore, the results are not generalised and are posited to pertain to only Sri Lankan users of Facebook, in addition to the other platforms and cultural environments”.* |
| The manuscript would benefit from a professional language edit to improve grammar, readability, and overall flow. | Thank you for this helpful suggestion. We have carefully revised the manuscript for grammar, readability, and overall flow, and also applied professional language editing to ensure clarity and consistency. |
